# Chronic Encapsulated Expanding Thalamic Hematoma Associated with Obstructive Hydrocephalus following Radiosurgery for a Cerebral Arteriovenous Malformation: A Case Report and Literature Review

**DOI:** 10.1155/2016/5130820

**Published:** 2016-01-24

**Authors:** Jun Takei, Toshihide Tanaka, Yohei Yamamoto, Akihiko Teshigawara, Satoru Tochigi, Yuzuru Hasegawa, Yuichi Murayama

**Affiliations:** ^1^Department of Neurosurgery, Jikei University School of Medicine Kashiwa Hospital, 163-1 Kashiwa-shita, Kashiwa, Chiba 277-8567, Japan; ^2^Department of Neurosurgery, Jikei University School of Medicine, 3-25-8 Nishi-Shinbashi, Minato-ku, Tokyo 105-8461, Japan

## Abstract

Chronic encapsulated intracerebral hematoma is a unique type of intracerebral hematoma accompanied by a capsule that is abundant in fragile microvasculature occasionally causing delayed regrowth. A 37-year-old man who had undergone radiosurgery for an arteriovenous malformation (AVM) causing intracerebral hematoma in the left parietal lobe presented with headache, vomiting, and progressive truncal ataxia due to a cystic lesion that had been noted in the left thalamus, leading to progressive obstructive hydrocephalus. He underwent left frontal craniotomy via a transsylvian fissure approach, and the serous hematoma was aspirated. The hematoma capsule was easy to drain and was partially removed. Pathological findings demonstrated angiomatous fibroblastic granulation tissue with extensive macrophage invasion. The concentration of vascular endothelial growth factor (VEGF) was high in the hematoma (12012 pg/mL). The etiology and pathogenesis of encapsulated hematoma are unclear, but the gross appearance and pathological findings are similar to those of chronic subdural hematoma. Based on the high concentration of VEGF in the hematoma, expansion of the encapsulated hematoma might have been caused by the promotion of vascular permeability of newly formed microvasculature in the capsule.

## 1. Introduction

Stereotactic radiosurgery (SRS) has become a therapeutic alternative for the treatment of cerebral arteriovenous malformations (AVMs). However, several delayed complications following SRS for AVMs including parenchymal hemorrhage, radiation necrosis, and cyst formation have been reported [1]. Among them, chronic encapsulated intracerebral hematoma is a rare cerebrovascular disease [1–8]. This type of hematoma expands slowly and behaves as a space-occupying lesion, sometimes resulting in obstructive hydrocephalus, uniquely located in the thalamus.

The thickened hematoma capsule possesses abundant microvasculature and can bleed easily when removed surgically, whereas the hematoma itself is serous and is usually easily aspirated. Therefore, the gross appearance and histological findings are similar to chronic subdural hematoma.

Vascular endothelial growth factor (VEGF), also known as vascular permeability factor (VPF), which promotes vascular permeability resulting in extravasation, is thought to be involved in the pathogenesis of chronic subdural hematoma [9, 10]. The role of VEGF in neovascularization and vascular hyperpermeability has been documented, confirming previous studies in which it has been stated that inflammation is responsible for angiogenesis of the hematoma capsule [9, 11, 12].

Surgical resection is the treatment of choice for improving neurological symptoms. Several different modalities of surgery have been suggested, including craniotomy, burr hole irrigation, and, in situations where the hematoma cavity is located near the ventricles causing obstructive hydrocephalus, endoscopic aspiration of the hematoma with fenestration.

## 2. Case Report

**Figure 1:**
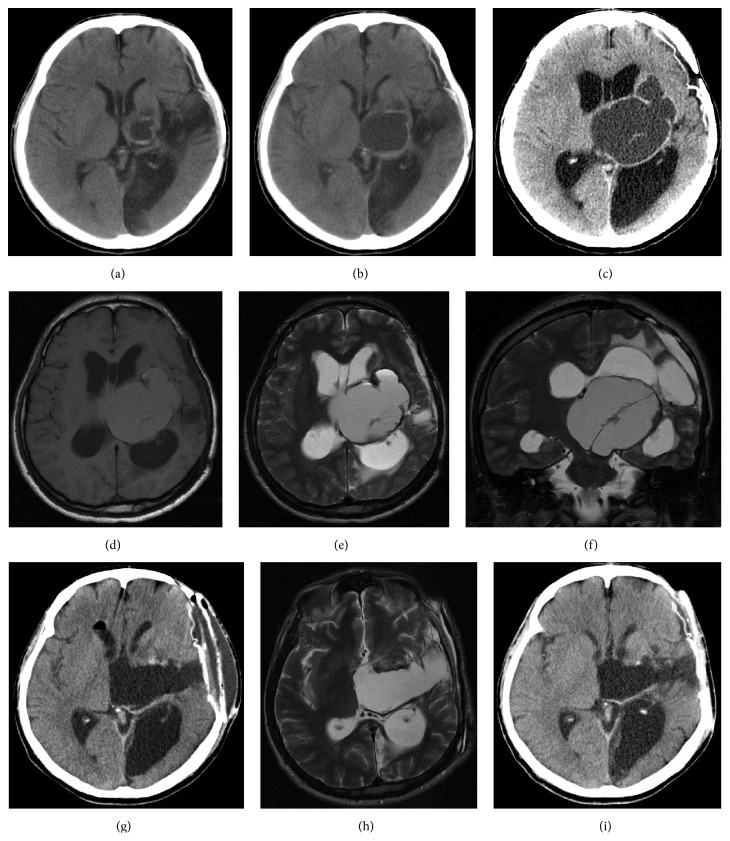
(a) Preoperative initial computed tomography (CT) 11 years after radiosurgery for an arteriovenous malformation in the left parietal lobe showing the cyst in the left thalamus. (b) Two years later, CT reveals that the size of the cyst is increased without hydrocephalus. (c) Four years later, CT reveals that the multilobular cyst is larger and accompanied by obstructive hydrocephalus. Preoperative magnetic resonance imaging (MRI) showing a cystic lesion in the left thalamus appearing isointense on T1-weighted imaging (d) and hyperintense on T2-weighted axial (e) and coronal (f) imaging as well as association with obstructive hydrocephalus. Note thickened cyst wall and septum in the middle of the cyst. Postoperative CT (g) and MRI (h) show shrinkage of the hematoma cavity and improvement of hydrocephalus. CT scan one year after surgery demonstrates neither recurrence of the hematoma nor progressive hydrocephalus (i).

A 37-year-old man presented with headache, vomiting, and progressive truncal ataxia. He had undergone radiosurgery and surgical extirpation for an arteriovenous malformation (AVM) causing intracerebral hematoma in the left parietal lobe 15 years prior to presentation. Since then, he had already been experiencing motor aphasia and right spastic hemiparesis. Eleven years after the radiosurgery for AVM, a cystic lesion with iso-low-density was noted in the left thalamus on computed tomography (CT) (Figure 1(a)). Initially, he was followed conservatively as he had no new neurological symptoms. The lesion was followed every other year and eventually revealed that the isodense cystic mass had grown gradually and was accompanied by progressive obstructive hydrocephalus (Figures 1(b) and 1(c)). Magnetic resonance imaging (MRI) showed a cystic lesion with a septum that appeared iso- and hyperintense on T1- and T2-weighted imaging, respectively (Figures 1(d)–1(f)).

Four years after initial CT, the patient underwent left frontal craniotomy via a trans-Sylvian fissure approach. The Sylvian fissure was dissected, and the lesion was identified. The cyst wall was incised and old serous hematoma was recognized. After aspiration of the hematoma, an elastic, hard, brownish-yellow cystic wall was partially removed because the hematoma capsule bled easily and hemostasis was very difficult to achieve, and then the foramen of Monro was identified. After the cerebrospinal fluid was drained, the swelling of the cerebral tissue resolved.

**Figure 2:**
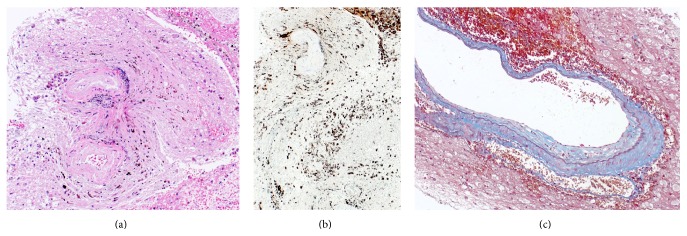
(a) Histological findings reveal hematoma capsule consisting of a dense collagenous layer with extensive invasion of macrophages, hematoxylin and eosin, ×40. (b) Immunohistochemical findings showing numerous CD68-positive cells in the cyst wall, original magnification ×40. (c) Vessel wall is thickened and internal elastica lamina is intact, revealing no residual nidus in the incised cyst wall, Masson, ×100.

Histological findings revealed that the hematoma wall consisted of an outer layer of dense collagenous tissue (Figures 2(a) and 2(c)) and an inner layer of angiomatous fibroblastic granulation tissue with extensive macrophage infiltration (Figure 2(b)).

Postoperatively, the patient's condition improved. Postoperative CT and MRI showed that the hematoma had been evacuated, and the hydrocephalus had improved (Figures 1(g) and 1(h)).

The concentration of VEGF quantified by ELISA (enzyme-linked immunosorbent assay) was 12012 pg/mL in the surgically excised hematoma. Neither further progression nor recollection of the hematoma was observed. He was transferred for further rehabilitation.

Follow-up CT scan one year after surgery demonstrated neither recurrence of the hematoma nor progressive hydrocephalus (Figure 1(i)).

## 3. Discussion

Chronic encapsulated intracerebral hematoma is a unique type of intracerebral hematoma first described by Hirch et al. and characterized by the presence of a fibrotic capsule [13]. Vascular anomalies such as AVM, cavernous angioma microaneurysm, and venous angioma are frequently seen. Thus, the pathogenesis of chronic encapsulated intracerebral hematoma is probably “self-destruction” or thrombosis during hemorrhagic episodes [14–16].

In recent years, chronic encapsulated hematoma has also been found to be associated with stereotactic radiosurgery for AVMs. Since Kurita et al. described an encapsulated intracerebral hematoma that developed after radiosurgery during the course of obliterating AVMs [1], 12 cases have been reported, including the present case (Table 1) [1–8]. Most of the lesions are located in the basal ganglia. Larger nidus volume and higher radiation dose may be a risk factor for delayed cyst formation [17]. The mean interval between radiosurgery for AVMs and surgical extirpation of chronic encapsulated hematoma was 6.5 years. Patient's average age was 34 years. In the series of encapsulated hematoma cases, most were accompanied by perifocal edema (Table 1).

Kurita et al. assumed that the cause of hematoma was probably repetitive bleeding from the fragile vessels contained in the thick hematoma capsule [1]. Chronic encapsulated intracerebral hematoma sometimes grows progressively while forming the capsule. However, the mechanism of the formation of chronic encapsulated hematoma still needs to be fully elucidated.

In contrast to previous reports, in the present case, CT demonstrated a low-density cystic lesion, indicating encapsulated hematoma in the chronic stage and progression of liquefied chronic hematoma. The AVM and encapsulated hematoma were situated apart from one another, and a residual nidus was not observed during surgery.

Potential mechanisms were considered for the development of a capsule after radiosurgery for AVMs histopathological findings demonstrated extensive microvasculature and suggested neovascularization in the densely collagenous capsule. Radiation necrosis was seen sporadically within the capsule as was active organization from fresh thrombus into hemosiderin within the fibrous tissue.

Bleeding and exudation from these fragile, newly formed vessels may have expanded the lesion in a fashion similar to chronic subdural hematoma.

Among the factors modulating angiogenesis, VEGF is one of the most likely candidates for a specific regulator that may promote the growth of this type of hematoma. VEGF, also known as VPF, is a potent mitogen for vascular endothelial cells and also promotes vascular permeability via the formation of vesiculovacuolar organelles in the cytoplasm of vascular endothelial cells [11, 12]. VEGF is thought to cause hypervascular tumor formation with expanding perifocal edema. In the same manner, a hematoma with a hypervascular capsule and perifocal edema might be caused by VEGF. The high concentration and expression of VEGF in the hematoma in the present case, similar to the previous report [6], as well as the microvascular endothelial proliferation in the hematoma capsule seen on immunohistochemical findings [5], suggest that angiogenesis and vascular permeability induced by VEGF might accelerate expansion of the hematoma.

Chronic encapsulated intracerebral hematoma often causes progressive neurological deficits due to mass effect. Two surgical approaches are typically considered: one is craniotomy via a trans-Sylvian route, and the other is an endoscopic approach with aspiration of the hematoma and cyst fenestration. As shown in Table 1, most cases have been treated by craniotomy. Only one case was treated by stereotactic aspiration of the hematoma followed by implantation of an Ommaya reservoir [6]. Since the chronic encapsulated intracerebral hematoma in our case had a tough membrane, separating the hematoma from the normal brain parenchyma was easy, as previously described [18]. We selected craniotomy; however, the capsule was very fragile and difficult to separate from the thalamus and bled easily. We were therefore only able to partially remove the hematoma capsule. Hemostasis was achieved by inserting cotton and thin-sliced Gelfoam soaked with fibrin glue. The hematoma was aspirated completely, whereas the capsule was only partially removed, which provided communication between the lateral ventricle and the third ventricle because the lesion was located in the thalamus and had led to obstructive hydrocephalus. For the reasons described above, in terms of hemostasis from the capsule of the hematoma, craniotomy was considered to be better than an endoscopic approach.

We believe that when the cystic hematoma revealed slow progression causing obstructive hydrocephalus, the patient should have undergone surgical resection earlier in order to obtain easier access and a more adequate working space. Moreover, closer long-term follow-up should be required for monitoring of the residual hematoma capsule. Radical resection of the capsule containing abundant neovasculature is obviously necessary to treat this type of hematoma; however, partial resection of the capsule is an alternative, especially in the case of a hematoma located in the thalamus accompanied by obstructive hydrocephalus.

## Figures and Tables

**Table 1:** Patients with chronic encapsulated intracerebral hematoma (CEIH) following radiosurgery for arteriovenous malformation.

Case number	Age	Sex	Location	Radiosurgery	Interval from radiosurgery to surgery (years)	Treatment of CEIH	Hematoma capsule	CT density	Edema	References
1	19	M	Rt. basal ganglia	GKS, 20 Gy	2	Craniotomy	Total removal	High	+	Kurita et al. 1996 [1]
2	51	M	Rt. basal ganglia	GKS, 22.5 Gy	6	Craniotomy	Total removal	High	+	Maruyama et al. 2006 [3]
3	47	M	Rt. caudate	GKS, 25 Gy	9	Craniotomy	Total removal	ND	+	Motegi et al. 2008 [4]
4	15	F	Rt. basal ganglia	LINAC, 15 Gy	7	Stereotactic aspiration	Not removed	High	−	Takeuchi et al. 2009 [6]
5	23	M	Rt. basal ganglia	GKS, 20 Gy	2	Craniotomy	Total removal	High	+	Nakamizo et al. 2011 [5]
6	57	M	Rt. basal ganglia	GKS, 22.5 Gy	5	Craniotomy	Total removal	High	+	Nakamizo et al. 2011 [5]
7	15	F	Rt. basal ganglia	GKS, 18 Gy	3	Craniotomy	Total removal	High	+	Nakamizo et al. 2011 [5]
8	55	M	Rt. frontal	LINAC, 20 Gy	11	Craniotomy	Total removal	ND	+	Nakamizo et al. 2011 [5]
9	49	M	Lt. basal ganglia	LINAC, 18 Gy	4	Craniotomy	Total removal	ND	+	Takeuchi et al. 2011 [7]
10	20	M	Rt. frontal	ND	10	Craniotomy	Total removal	ND	+	Lee et al. 2011 [2]
11	20	F	Lt. cerebellar	GKS, 20 Gy	4	Craniotomy	Total removal	High	+	Watanabe et al. 2014 [8]
12	37	M	Lt. thalamus	ND	15	Craniotomy	Partial removal	Iso	+	Present case

F: female; M: male; Lt.: left; Rt.: right; GKS: gamma knife surgery; LINAC: linear accelerator radiosurgery; ND: not described.

## References

[1] Kurita H., Sasaki T., Kawamoto S. (1996). Chronic encapsulated expanding hematoma in association with gamma knife stereotactic radiosurgery for a cerebral arteriovenous malformation: case report. *Journal of Neurosurgery*.

[2] Lee C.-C., Pan D. H.-C., Ho D. M.-T. (2011). Chronic encapsulated expanding hematoma after gamma knife stereotactic radiosurgery for cerebral arteriovenous malformation. *Clinical Neurology and Neurosurgery*.

[3] Maruyama K., Shin M., Tago M. (2006). Management and outcome of hemorrhage after gamma knife surgery for arteriovenous malformations of the brain. *Journal of neurosurgery*.

[4] Motegi H., Kuroda S., Ishii N. (2008). De novo formation of cavernoma after radiosurgery for adult cerebral arteriovenous malformation—case report. *Neurologia Medico-Chirurgica*.

[5] Nakamizo A., Suzuki S. O., Saito N. (2011). Clinicopathological study on chronic encapsulated expanding hematoma associated with incompletely obliterated AVM after stereotactic radiosurgery. *Acta Neurochirurgica*.

[6] Takeuchi S., Takasato Y., Masaoka H. (2009). Development of chronic encapsulated intracerebral hematoma after radiosurgery for a cerebral arteriovenous malformation. *Acta Neurochirurgica*.

[7] Takeuchi S., Takasato Y., Masaoka H. (2011). Chronic encapsulated intracerebral hematoma formation after radiosurgery for cerebral arteriovenous malformation. *Neurology India*.

[8] Watanabe T., Nagamine H., Ishiuchi S. (2014). Progression of cerebellar chronic encapsulated expanding hematoma during late pregnancy after gamma knife radiosurgery for arteriovenous malformation. *Surgical Neurology International*.

[9] Hohenstein A., Erber R., Schilling L., Weigel R. (2005). Increased mRNA expression of VEGF within the hematoma and imbalance of angiopoietin-1 and -2 mRNA within the neomembranes of chronic subdural hematoma. *Journal of Neurotrauma*.

[10] Vaquero J., Zurita M., Cincu R. (2002). Vascular endothelial growth-permeability factor in granulation tissue of chronic subdural haematomas. *Acta Neurochirurgica*.

[11] Dvorak A. M., Kohn S., Morgan E. S., Fox P., Nagy J. A., Dvorak H. F. (1996). The vesiculo-vacuolar organelle (VVO): a distinct endothelial cell structure that provides a transcellular pathway for macromolecular extravasation. *Journal of Leukocyte Biology*.

[12] Dvorak H. F., Brown L. F., Detmar M., Dvorak A. M. (1995). Vascular permeability factor/vascular endothelial growth factor, microvascular hyperpermeability, and angiogenesis. *American Journal of Pathology*.

[13] Hirsh L. F., Spector H. B., Bogdanoff B. M. (1981). Chronic encapsulated intracerebral hematoma. *Neurosurgery*.

[14] Fiumara E., Gambacorta M., D'Angelo V., Ferrara M., Corona C. (1989). Chronic encapsulated intracerebral haematoma: pathogenetic and diagnostic considerations. *Journal of Neurology Neurosurgery and Psychiatry*.

[15] Pozzati E., Giuliani G., Gaist G., Piazza G., Vergoni G. (1986). Chronic expanding intracerebral hematoma. *Journal of Neurosurgery*.

[16] Sakaida H., Sakakura M., Tochio H., Nakao K., Taniguchi A., Yabana T. (1993). Chronic encapsulated intracerebral hematoma associated with angiographically occult arteriovenous malformation—case report. *Neurologia Medico-Chirurgica*.

[17] Izawa M., Hayashi M., Chernov M. (2005). Long-term complications after gamma knife surgery for arteriovenous malformations. *Journal of Neurosurgery*.

[18] Nishiyama A., Toi H., Takai H. (2014). Chronic encapsulated intracerebral hematoma: three cases reports and a literature review. *Surgical Neurology International*.

